# Bim Mediates the Elimination of Functionally Unfit Th1 Responders from the Memory Pool

**DOI:** 10.1371/journal.pone.0067363

**Published:** 2013-06-28

**Authors:** David C. Jay, Diana M. Mitchell, Matthew A. Williams

**Affiliations:** Department of Pathology, University of Utah, Salt Lake City, Utah, United States of America; University of Iowa, United States of America

## Abstract

Selective clonal deletion in the CD4^+^ T cell compartment during the transition from effector to memory is accompanied by enhanced expression of the pro-apoptotic Bcl-2 family member Bim. Here, we show that Bim deficiency enables the survival of poorly functional Th1 responders that are normally eliminated during contraction. However, rescued *bim* ^−/−^ CD4^+^ “memory” T cells continued to demonstrate deficient effector functions, poor sensitivity to antigen and an inability to respond to secondary challenge. Our results demonstrate that Bim activity plays a key role in shaping the CD4^+^ memory T cell repertoire, ensuring the emergence of highly functional CD4^+^ memory T cells and the elimination of Th1 effector cells with sub-optimal function. We propose that Bim is a key mediator of T cell death in the absence of appropriate TCR-driven activation and differentiation.

## Introduction

Following acute viral or bacterial infection, antigen-specific T cells clonally expand and acquire effector functions that contribute to pathogen clearance. The expansion phase is robust, representing as much as a 50,000–100,000-fold increase from the naïve precursor frequency. Following elimination of the pathogen, 90–95% of effector T cells die, leaving behind a long-lived pool of memory T cells. These memory T cells provide protection to subsequent infections with the same or similar pathogens [1]. A major focus of recent study is to determine the molecular signals that control the memory fate decision allowing a minority of effector T cells to survive into the memory pool.

CD4^+^ T cell responses to acute viral or intracellular bacterial infection differ from CD8^+^ T cell responses with respect to their requirement for TCR-driven differentiation signals. For example, CD8^+^ T cells require only a short period of antigenic stimulation (6–24 hours) to drive recruitment and programmed differentiation [2]–[5]. CD4^+^ T cells, on the other hand, require several days of in vivo antigen exposure to achieve maximal expansion and differentiation into pathogen-specific effector cells [6]–[8]. These observations suggest that CD4^+^ T cells translate TCR signals into a downstream activation/differentiation program in a fundamentally different fashion than do CD8^+^ T cells. Additionally, the requirement for prolonged exposure to antigen suggests that CD4^+^ T cell differentiation and survival may be prone to selection on the basis of qualitative and/or quantitative components of the antigen signal [9]–[11].

One insight into these potential differences between CD4^+^ and CD8^+^ T cells came from the observation that CD8^+^ T cell repertoires are unchanged after the effector phase. For CD8^+^ T cells, there is no difference in the frequency of distribution of individual clonal populations within a pool of cells with a given specificity when comparing effector, memory or secondary effector populations [12]. Conversely, CD4^+^ T cell repertoires undergo antigen-driven revision and skewing during successive antigen challenges [9], [13]. However, one important issue unresolved by these prior studies is how the differentiation and survival of effector cells during the transition into the Th1 memory pool is dependent upon TCR-mediated signals delivered during the primary response, including the amount and duration of antigen, structural avidity of the TCR, the type and maturation state of the antigen presenting cell, and the inflammatory environment.

CD4^+^ T cell clones that populate the Th1 effector pool do not compete equally for entry into the memory compartment. Following infection with lymphocytic choriomeningitis virus (LCMV), small numbers of adoptively transferred SMARTA TCR transgenic T cells, which are specific for a LCMV glycoprotein epitope (GP_61–80_), responded in a manner that mirrored the functionality, kinetics, effector differentiation, and memory development of polyclonal endogenous CD4^+^ responders to the same peptide in the same host. Conversely, following infection with a *Listeria monocytogenes* engineered to secrete the LCMV GP_61–80_ epitope (Lm-gp61), SMARTA cells developed sub-optimal effector function as compared to polyclonal endogenous CD4^+^ T cell responders to the same epitope in the same host, exemplified by decreased antigen sensitivity and lower cytokine production, and failed to populate the memory pool [14]. Lm-gp61 itself is not defective in its ability to stimulate Th1 memory, as endogenous primary and secondary Th1 memory cells are readily detectable up to a year post-infection [14], [15]. Specifically, it was the SMARTA TCR transgenic T cells that are defective in their ability to enter the memory pool in the context of the Lm-gp61 infection. Our previous findings have found that SMARTA cells display defective functional avidity prior to their disappearance, and our extensive analysis of both primary and secondary CD4 memory development has found a strong correlation between functional avidity [14], as calculated by measuring IFNγ production in response to decreasing concentrations of peptide during ex vivo restimulation, and the likelihood of entering the memory pool. These observations have led us to seek to determine the mechanisms regulating the elimination of SMARTA cells in this setting. Because SMARTA cells are monoclonal, we hypothesized that quality and duration of signaling during the primary response may play a role in the specification of CD4^+^ memory T cell fate [14].

The downstream molecular pathways that link signal strength during the primary response to survival into the CD4^+^ T cell memory pool are not well understood. We observed that SMARTA effector cells exhibited increased expression of Bim mRNA transcripts at the peak of the response to Lm-gp61, as compared to SMARTA effector cells induced by LCMV. Bim is a pro-apoptotic BH3-only Bcl-2 family member that promotes apoptosis by directly or indirectly inhibiting anti-apoptotic Bcl-2 [16]. Bim regulates T cell survival during several stages of T cell development and differentiation [17], [18]. The relative balance of Bim and Bcl-2 activity in any given T cell is thought to be a key determinant of survival during thymic selection and in mature peripheral T cells [19]. Of particular relevance, Bim has been shown to mediate the loss of effector CD4^+^ and CD8^+^ T cells following antigen clearance during the contraction phase of the T cell response to several pathogenic infections [20]–[24]. However, the extrinsic and intrinsic signals that regulate Bim activity during the acute response to infection have not been well defined.

Due to its known role in contraction, we hypothesized that increased Bim activity during the primary response accounted for the elimination of SMARTA cells following infection with Lm-gp61. To address this problem experimentally, we crossed SMARTA mice to a Bim-deficient (*bim^−/−^*) background and co-transferred small numbers of wildtype and *bim^−/−^* SMARTA cells into the same host prior to Lm-gp61 infection. Simultaneously tracking wildtype (WT) and *bim^−/−^* SMARTA cells, we found that both populations expanded similarly following Lm-gp61 infection. As previously observed, WT SMARTA cells disappeared in the weeks following pathogen clearance. In contrast, *bim^−/−^* SMARTA cells successfully populated the memory pool, although they lacked several memory CD4^+^ T cell functional characteristics when compared to polyclonal memory CD4^+^ T cells directed towards the same epitope. More specifically, “memory” *bim* ^−/−^ SMARTA cells were poor producers of the effector cytokines IFNγ, TNFα and IL-2, and they failed to generate a secondary response to homologous or heterologous rechallenge. These findings demonstrate an obligate role for Bim in preventing the entry of poorly functional SMARTA effector Th1 cells into the memory pool and suggest that one consequence of memory differentiation signals during the effector response is to modulate Bim activity. Bim therefore acts as a means to prevent the formation of poorly functional CD4^+^ memory T cells that are unlikely to successfully participate in a secondary response.

## Methods

### Ethics Statement

This study was carried out in accordance with the recommendations provided by the Guide for the Care and Use of Laboratory Animals of the National Institutes of Health. This study was approved by the University of Utah Animal Care and Use Committee (PHS Assurance Registration Number A3031-01, Protocol Number 12-10011).

### Mice and Infections

C57BL/6 (B6) and *bim^−/−^* mice on a B6 genetic background were purchased from Jackson Laboratories (Bar Harbor, ME). SMARTA TCR transgenic mice [25] were maintained in SPF facilities at the University of Utah. Lymphocytic choriomeningitis virus (LCMV) Armstrong 53b and recombinant vaccinia virus was grown and titered as previously described [26], [27]. For primary challenges and heterologous rechallenges, mice were infected i.p. with 2×10^5^ plaque-forming units (PFU) LCMV or 2×10^6^ PFU recombinant vaccinia virus expressing the full length LCMV glycoprotein (Vac-GP) [28], or i.v. with 2×10^5^ colony-forming units (CFU) recombinant *Listeria monocytogenes* (Lm-gp61) (a gift from M. Kaja-Krishna, University of Washington, Seattle, WA). Lm-gp61 was prepared as previously described [14]. For homologous secondary challenges with Lm-gp61, mice were injected i.v. with 1×10^6^ CFU.

### Adoptive Transfers

Splenocyte cell suspensions were generated from SMARTA mice and untouched CD4^+^ T cells were isolated using magentic beads per manufacturer’s instructions (Miltenyi Biotec, Auburn, CA), but with the addition of biotinylated anti-CD44 antibody (eBiosciences, San Diego, CA) to mediate the removal of memory phenotype cells. SMARTA cell purity and phenotype was assessed by flow cytometric analysis. SMARTA cells (5×10^3^) were re-suspended in PBS and injected i.v. into recipient mice one day prior to infection.

### Mixed Bone Marrow Chimeras

B6 (Thy1.2^+^CD45.2^+^) mice were lethally irradiated with two doses of 450 rads separated by several hours using the x-irradiatior in the mouse vivarium at the University of Utah. One day later, mice received a 1∶1 mix of 5×10^6^ bone marrow cells harvested from the femurs and tibias of donor mice as indicated. Bone marrow cells were prepared by red blood cell lysis and depletion of CD3^+^ T cells using biotinylated anti-CD3 antibodies (eBioscience, San Diego, CA) and magnetic beads (Miltenyi Biotec, Auburn, CA) per manufacturer’s instructions. After 8–10 weeks, reconstitution was assessed using antibodies to the Thy1.1 and CD45.1 congenic markers.

### Antibodies and Flow Cytometry

Cell surface stains were done in PBS containing 1% FBS and 2 mM EDTA with fluorescently labeled antibodies to CD4, Thy1.1, Thy1.2, and Vα2 (eBiosciences, San Diego, CA; Biolegend, San Diego, CA). For staining with anti-Bcl-2 (BD Biosciences, San Diego, CA) or anti-Bim antibodies (Cell Signaling Technology, Danvers, MA), cells were stained with cell-surface antibodies and then permabilized with Cytofix/Cytoperm buffers per manufacturer’s instructions (BD Biosciences, San Diego, CA). Anti-Bim was visualized using a fluorescently-tagged secondary goat anti-rabbit antibody (Invitrogen). Flow cytometry was performed using a FACSCanto II or LSRFortessa flow cytometer (BD Biosciences) and analyzed using FlowJo (Treestar, Mountain View, CA).

### Ex vivo Restimulation and Intracellular Cytokine Staining

Splenocyte cell suspensions (2×10^6^) in DMEM supplemented with 10% fetal bovine serum, antibiotics and L-glutamine were restimulated for 4 hours with 1 µM (or titrated dilutions) GP_61–80_ peptide from LCMV (GLKGPDIYKGVYQFKSVEFD) in the presence of Brefeldin A (GolgiPlug, 1 µl/ml), per manufacturers instructions (BD Biosciences). Following restimulation, cells were stained with fluorescently-labeled cell surface antibodies, permeabilized, stained intracellularly with fluorescently-labeled antibodies to IFNγ, TNFα, and IL-2 using a Cytofix/Cytoperm kit per manufacturers instructions (BD Biosciences), and then analyzed by flow cytometry.

## Results

### Bim Expression is Upregulated in Lm-gp61-induced SMARTA Effector Th1 Cells

**Figure 1:**
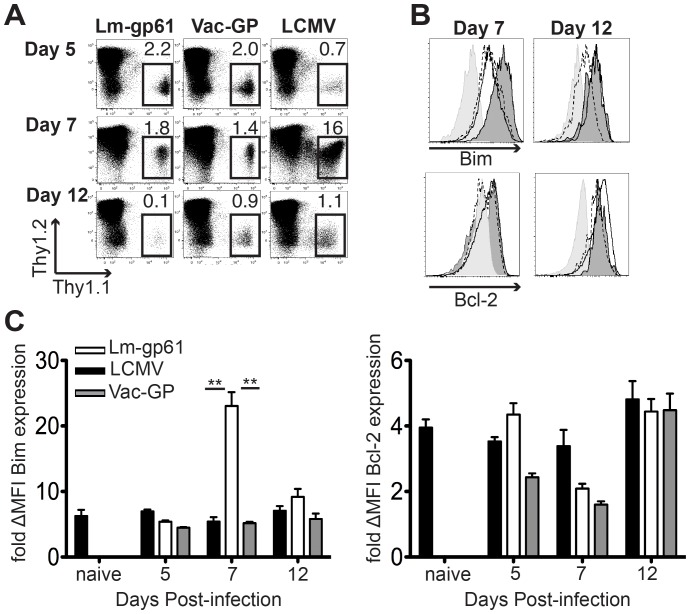
Bim expression is up-regulated in Lm-gp61-induced SMARTA effector Th1 cells. We transferred 1×10^4^ CD44^lo^ SMARTA cells (Thy1.1^+^) into naïve B6 hosts and infected with Lm-gp61, LCMV or Vac-GP on day later. *A*, Representative flow plots indicate the frequency of SMARTA cells in the spleen at days 5, 7 and 12 post-infection. *B*, Representative histograms indicate expression of Bim or Bcl-2 by SMARTA cells following infection with LCMV (solid line), Vac-GP (dashed line) or Lm-gp61 (dark gray fill) as compared to isotype controls (light gray fill) at the indicated time points post-infection. *C*, Bar graph displays the fold shift in Bim or Bcl-2 mean fluorescence intensity (MFI) as compared to isotype controls in SMARTA cells from naïve mice or at each time point post-infection. Plots represent 3–4 mice/group and results are representative of two independent experiments. Error bars indicate the standard error of the mean (SEM). *p* values for statistically significant differences were calculated by a two-tailed Student’s T test. **p≤0.01, *p≤0.05.

We previously found that SMARTA effector Th1 cells generated following Lm-gp61 infection expressed higher levels of Bim mRNA transcripts at the peak of their response compared to SMARTA effector Th1 cells induced by LCMV infection. Increased Bim transcripts corresponded with a failure to populate the memory compartment [14]. In order to better understand the role of Bim we assessed Bim protein expression in SMARTA cells following infection with Lm-gp61, LCMV or recombinant vaccinia virus expressing LCMV glycoprotein (Vac-GP) by flow cytometry. As expected, there is a rapid culling of the SMARTA cells during the contraction phase following an Lm-gp61 infection (Fig. 1A). At the peak of the effector response (day 7), Lm-gp61-induced SMARTA effector cells expressed several-fold higher levels of Bim compared to SMARTA effector cells following infection with LCMV or Vac-GP (Fig. 1B and 1C), as well as compared to naïve SMARTA cells. It has previously been reported that the balance of Bim and Bcl-2 is critical for maintaining survival of T cells [19]. The significant increase in Bim expression among Lm-gp61-induced SMARTA cells at the peak of the effector response occurs while Bcl-2 expression remains similar (Fig. 1B–C). During the priming and contraction phases (days 5 and 12, respectively), Bim was expressed at roughly similar levels, regardless of the infection (Fig. 1B–C).

### Bim Mediates the Elimination of SMARTA Cells Following Lm-gp61 Infection

**Figure 2:**
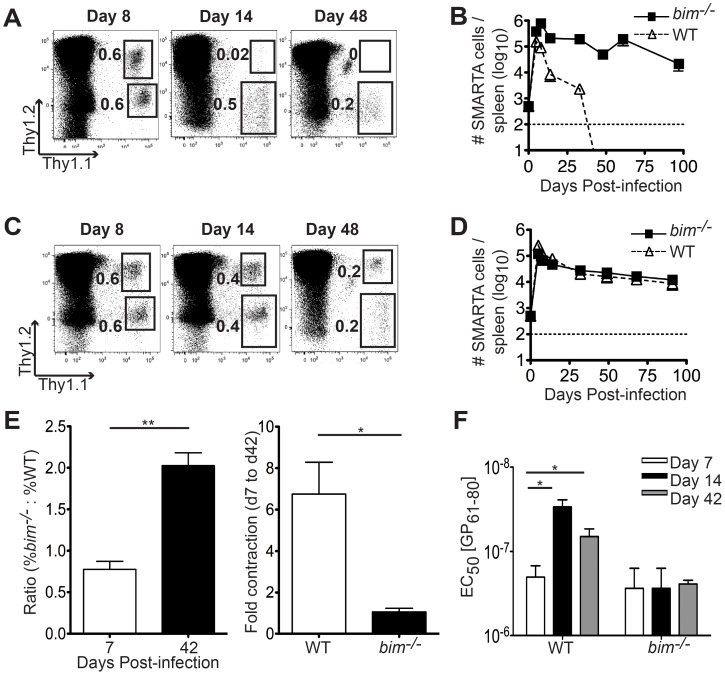
Bim mediates the elimination of SMARTA cells following Lm-gp61 infection. We co-transferred 5×10^3^ each WT SMARTA (Thy1.1^+^ Thy1.2^+^) and *bim* ^−/−^ SMARTA (Thy1.1^+^) into B6 hosts (Thy1.2^+^), followed by infection with either Lm-gp61 or Vac-GP one day later. *A* and *C*, Representative plots indicate expansion and survival of SMARTA cells in the spleen following Lm-gp61 or Vac-GP infection. *B* and *D*, Graph indicates the survival of WT or *bim* ^−/−^ SMARTA cells in the spleen following Lm-gp61 infection. Dashed line indicates the limit of detection. Results are representative of 3–5 mice per group per time point and four independent experiments. *E*, Mixed bone marrow chimeras, generated using a 1∶1 mix of wildtype (CD45.1^+^) and Bin-deficient (Thy1.1^+^) bone marrow injected into lethally irradiated B6 (Thy1.2^+^CD45.2^+^) hosts, were infected with Lm-gp61 8–10 weeks post-transplant. The number of IFNγ-producing Th1 effector or memory cells in the spleen was determined at 7 or 42 days post-transplant. *F*, Splenocytes harvested at the indicated time points were stimulated with decreasing concentration of GP_61–80_ peptide for four hours *ex vivo* in the presence of Brefeldin A, followed by intracellular antibody staining for IFNγ. Bar graphs indicate the effective peptide concentration required to elicit the half maximal response. Error bars indicate the SEM (n = 4 mice/group at each time point). *p* values for statistically significant differences were calculated by a two-tailed Student’s T test. **p≤0.01, *p≤0.05.

To determine if Bim is required to promote the death of dysfunctional Lm-gp61-induced SMARTA cells, we crossed SMARTA mice to a *bim^−/−^* background. Mice were bred to congenic backgrounds so that *bim* ^−/−^ SMARTA cells (Thy1.1^+^Th1.2^+^) could be co-transferred with littermate control WT SMARTA (Thy1.1^+^) into naive B6 hosts (Thy1.2^+^), followed by infection with Lm-gp61 one day later. Following Lm-gp61 infection, WT SMARTA cells expanded, contracted and disappeared from the memory pool within a few weeks, as previously reported [14]. However, *bim* ^−/−^ SMARTA survived into the memory phase with kinetics similar to polyclonal endogenous CD4^+^ T cell responders to the same epitope in the same host (Fig. 2A and 2B, data not shown). Following Vac-GP infection, WT and *bim* ^−/−^ SMARTA efficiently populated the memory pool with similar efficiency, indicating that the unique role of Bim in regulating cell death of low avidity Lm-gp61-induced SMARTA cells during the contraction phase may rely in part on the nature of the infectious challenge (Fig. 2C and 2D). No differences were observed in the differentiation of central or effector memory populations, or the expression of activation or localization markers between WT and *bim^−/−^* SMARTA populations during Vac-GP infection (data not shown). It is important to note that in contrast to other infectious models [22], [24], Bim deficiency did not impart a survival advantage to SMARTA cells during the contraction phase following Vac-GP infection, indicating that the role of Bim may vary depending on the infectious model.

Similar experiments were done with LCMV. However, when either *bim^−/−^* or *bim^+/−^* SMARTA cells were co-transferred with littermate control *bim^+/+^* SMARTA cells, they disappeared within 4 weeks post-infection (data not shown). These findings suggested that transplanted SMARTA cells containing the “knock-out” allele were rejected following LCMV infection, possibly due to linkage to a minor histocompatibility locus located near the *bim* locus [29]. These observations pertained only to the LCMV-infection model, and not to the Lm-gp61 and Vac-GP infectious model systems. Therefore, our future studies focused on these two infectious model systems.

One possible drawback to the use of transgenic T cells is the possibility that they may not be completely representative of the endogenous response. Therefore, we established a system for the analysis of endogenous Th1 responses to Lm-gp61 infection. We generated mixed bone marrow chimeras in which lethally irradiated B6 hosts (Thy1.2^+^, CD45.2^+^) were rescued with a 1∶1 mixture of bone marrow from wildtype (CD45.1^+^) and Bim-deficient (Thy1.1^+^) donors. Because of the combination of CD45 and Thy1 congenic alleles, we were able to readily detect wildtype and Bim-deficient donor T cells 8–10 weeks later. The use of mixed bone marrow chimeras allowed us to assess the CD4^+^ T cell intrinsic role of Bim, as well as control for potential differences in pathogen clearance. Following Lm-gp61 infection, we observed the generation of both wildtype and Bim-deficient Th1 cells in the spleen at the peak of the effector response (day 7). However, while wildtype Th1 effector cells contracted substantially (∼7-fold) during the transition to memory between days 7 and 42 post-infection, Bim-deficient responders underwent virtually no contraction (Fig. 2E). Additionally, the emergence of Th1 memory cells within the wildtype population was accompanied by an overall increase in functional avidity, as we have previously reported [14]. In contrast, Bim-deficient memory cells maintained the low functional avidity characterized by the effector response (Fig. 2F), suggesting that in the absence of Bim poorly functional Th1 responders failed to be eliminated. These findings indicate a key role for Bim in shaping the functional memory Th1 repertoire.

### Persisting *bim^−/−^* SMARTA “Memory” Cells are Functionally Defective

**Figure 3:**
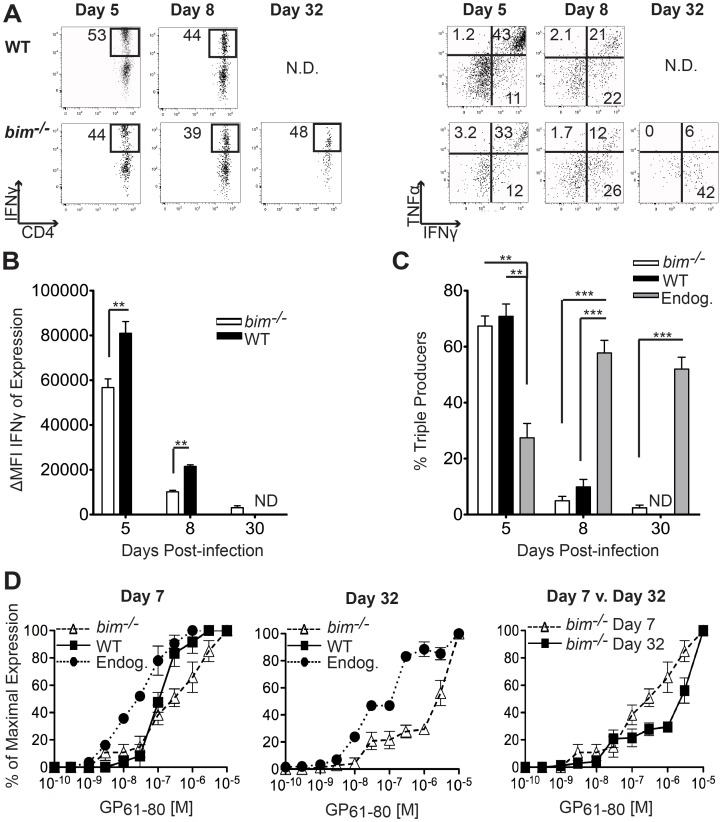
Persisting *bim^−/−^* SMARTA “memory” cells are functionally defective. We analyzed the functionality of SMARTA responses in the spleen following Lm-gp61 infection. *A*, Representative plots indicate the expression of IFNγ and TNFα by WT or *bim* ^−/−^ SMARTA cells in the spleen at the indicated time points after infection with Lm-gp61. *B*, Bars graph indicate the shift in MFI of IFNγ-producing cells, as compared to unstimulated controls. *C*, Bar graph indicates the percent of IFNγ-producing SMARTA cells that also make TNFα and IL-2 (“triple producers”). *D*, Graphs display the frequency of IFNγ-producing SMARTA cells or polyclonal endogeneous CD4^+^ T cells specific for the same epitope over a range of peptide concentrations as a percentage of the maximal response (defined as the response at the highest peptide concentration). Results are representative of 3–5 mice per group per time point and four independent experiments. Error bars indicate the SEM.

The ability to produce multiple cytokines (i.e. TNFα and IL-2) and high levels of IFNγ have been correlated with the quality of the CD4^+^ T cell memory pool and enhanced protective function [30], [31]. Our prior studies found that SMARTA effector cells generated following Lm-gp61 infection demonstrated poor function as measured by the frequency of responders able to produce IFNγ, IL-2 and TNFα simultaneously upon restimulation and the amount of cytokine produced on a per cell basis [14]. We therefore determined whether Bim-deficiency could rescue effector function along with the survival of SMARTA cells following Lm-gp61 infection. Despite their enhanced survival, *bim^−/−^* SMARTA cells demonstrated consistently poor functionality throughout the effector and memory phases following Lm-gp61 infection, largely solely producing IFNγ (Fig. 3A and C). At effector time points following Lm-gp61 infection, both WT and *bim^−/−^* SMARTA cells were capable of making IFNγ upon restimulation (Fig. 3A). Similarly, in the early stages of the contraction phase, while wildtype SMARTA cells were still detectable (up to day 15), both WT and *bim* ^−/−^ SMARTA cells produced IFNγ upon restimulation (data not shown). However, at all time points tested they produced much less on a per cell basis than did the polyclonal endogenous responders to the same epitope (Fig. 3B, data not shown). Furthermore, surviving *bim* ^−/−^ SMARTA memory cells were poor producers of multiple cytokines (IFNγ, IL-2, TNFα) (Fig. 3C).

Others and we have reported that both SMARTA and polyclonal effector Th1 cells acquire higher functional avidity (sensitivity to antigenic stimulation leading to a functional response, i.e. IFNγ production) throughout the primary response and as they transition into the memory pool [14], [32]. Similar to what we have previously reported for WT SMARTA cells [14], at the peak of the effector response *bim* ^−/−^ SMARTA memory cells possessed a functional avidity lower than the polyclonal endogenous CD4^+^ response to the same epitope (Fig. 3D). Because the formation of highly functional, long-lived memory populations corresponds to the emergence of high functional avidity memory cells, we directly compared the functional avidity of effector (d7) and memory (d32) SMARTA Th1 cells. Strikingly, the transition to memory resulted in no functional avidity maturation. Instead, the low functional avidity of *bim^−/−^* SMARTAs was maintained at memory time points (Fig. 3D), showing that merely enabling the survival of CD4^+^ effector Th1 populations into the memory compartment does not ensure the acquisition of memory function. Thus, following infection with a particular pathogen, Bim can promote CD4^+^ T cell *survival* during the transition to memory, but the development of memory *function* is Bim-independent, as evidenced by the survival of Bim-deficient SMARTA memory cells that were profoundly dysfunctional.

### *bim* ^−/−^ SMARTA “Memory” Cells Lack the Ability to Respond to Secondary Challenge

**Figure 4:**
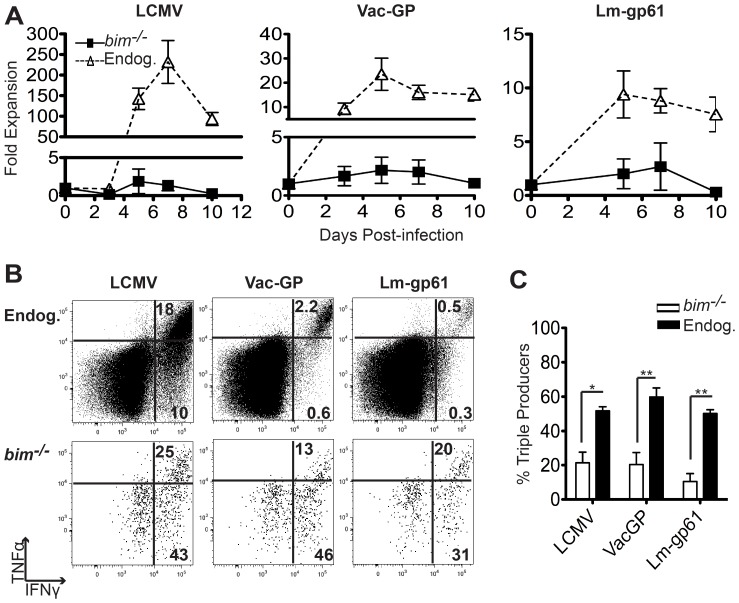
*bim* ^−/−^ SMARTA “Memory” cells lack the ability to respond to secondary challenge. Lm-gp61 immune mice (day 90 post-infection) containing “memory” *bim* ^−/−^ SMARTA were rechallenged with LCMV, Vac-GP or Lm-gp61 and the endogenous and *bim* ^−/−^ SMARTA responses to GP_61–80_ were assessed. *A*, Graphs display fold expansion of SMARTA cells or IFNγ-producing polyclonal endogenous CD4^+^ T cells at the indicated time points post-rechallenge. *B*, Representative flow plots display the cytokine production profile of polyclonal endogenous CD4^+^ T cells and *bim* ^−/−^ SMARTA cells following *ex vivo* peptide restimulation at day 5 post-rechallenge. *C*, Bar graph indicates the percent of IFNγ-producing *bim* ^−/−^ SMARTA cells and polyclonal endogenous IFNγ-producing cells that also make TNFα and IL-2 (“triple producers”). Results are representative of 4–5 mice per group per time point and two independent experiments. Error bars indicate the SEM.

To directly test their memory function, we rechallenged Lm-gp61-generated *bim* ^−/−^ SMARTA memory cells either homologously with Lm-gp61 or heterologously with LCMV or Vac-GP. Whether rechallenged with Lm-gp61, Vac-GP or LCMV, *bim* ^−/−^ SMARTA memory cells failed to significantly expand as compared to the endogenous memory cells in the same host (Fig. 4A). Similarly, at day 5 post-rechallenge, *bim* ^−/−^ SMARTA memory cells demonstrated consistently poor effector function, as measured by their ability to make multiple cytokines upon restimulation (IFNγ, TNFα and IL-2). *bim* ^−/−^ SMARTA secondary responders continued to be largely comprised of IFNγ mono-producers, in sharp contrast to the multiple cytokine production of polyclonal endogenous secondary responders (Fig. 4B and C). This dysfunctional phenotype was maintained throughout the course of the recall response (data not shown).

## Discussion

Overall, our findings demonstrate that Bim itself is capable of intrinsically mediating the death of functionally defective, low avidity SMARTA effector Th1 cells generated following Lm-gp61 infection. *bim* ^−/−^ SMARTA cells were able to survive beyond the effector phase and maintain themselves similarly to endogenous responders in the same host, yet they failed to acquire the functional characteristics of a memory population. *bim^−/−^* SMARTA cells demonstrated and maintained poor effector function when restimulated with peptide and failed to mount substantial *in vivo* recall responses following rechallenge. Thus, while Bim is required to regulate the survival of poorly functional SMARTA cells following Lm-gp61 infection, it alone is not sufficient to restore their ability to become fully functional memory cells. One caveat to the use of SMARTA transgenic T cells is the possibility that they are not representative of polyclonal endogenous Th1 effector and memory cells. Our studies of endogenous Bim-deficient CD4^+^ T cells, however, similarly suggest that the absence of contraction by Bim-deficient T cells corresponds to the rescue and entry of memory cells into the memory pool with poor functional avidity. Overall, our results highlight a key function for Bim in functionally shaping the Th1 memory repertoire.

While Bim has been found to have a role in mediating activated T cell contraction after antigen clearance following infection with certain pathogens, the signals that lead to Bim-mediated apoptosis in most CD4^+^ T cells but not those fated to enter the memory pool remain unknown. Our prior findings indicated that Bim expression was clonally selective, depending on the infectious model. In those prior studies, the differential ability of LCMV- or Lm-gp61-induced SMARTA effector Th1 cells to survive into the memory pool corresponded strongly (and inversely) with the expression of Bim transcripts [14]. Here we show a required mechanistic role for Bim in the elimination of dysfunctional SMARTA Th1 cells induced by Lm-gp61. Because these are monoclonal populations, one possibility is that Bim activity, and subsequent Bim-regulated survival, are influenced by the qualitative or quantitative nature of the TCR-mediated activation signal during primary activation.

Little is known about how the nature or timing TCR signals may influence the decision of a CD4^+^ T cell to enter a Bim-mediated cell death pathway. Previous work from our lab has shown that by as early as day five post Lm-gp61 infection, “doomed to die” SMARTA cells cannot be recovered by a subsequent LCMV infection [14]. Thus, merely increasing the presence of antigen in a context that normally stimulates SMARTA memory formation does not rescue the survival or functionality of Lm-gp61-induced SMARTA effector cells. Instead, the decision to enter a Bim-dependent apoptotic pathway likely occurs early in the priming phase, well before the observed up-regulation of Bim expression. We did not observe significant up-regulation of Bim expression in SMARTA cells until the peak of the effector response to Lm-gp61 (day 7 post-infection). We hypothesize that the up-regulation of Bim is a consequence of the qualitative nature of TCR activation signals received early during the priming phase, such that Bim expression serves as a sensor of the fitness of a CD4^+^ T cell clone to enter the memory pool but not a key mediator of functionally defective CD4^+^ effector T cell responses. Importantly, Bim has been shown to promote death of functionally fit Th1 effector cells as well [22], [24], indicating that Bim activity and subsequent memory T cell differentiation can be influenced by both T cell-intrinsic (i.e. TCR-mediated activation) and extrinsic signals. Additionally, others have shown that Bim can promote the death of functionally protective responders in settings of chronic infection, reflecting the complex nature of the magnitude and duration of signaling in dictating T cell fate specification [23], [24].

Although TCR signaling can regulate Bim expression in immature thymocytes, the factors upstream of Bim that may connect its expression to TCR and inflammatory environment signaling are not well understood [33]. One possible candidate is Foxo3a, a transcription factor that regulates the expression of several cell cycle inhibitors and proapoptotic factors, including Bim, and is upregulated in Lm-gp61-induced SMARTA cells [14]. Foxo3a-deficient mice have increased T cell accumulation and magnitude of expanded antigen specific T cells following LCMV infection, but it is debatable whether this is dependent upon T cell intrinsic defects, or extrinsic defects in dendritic cell IL-6 signaling that allows increased T cell viability [34]–[36]. Studies have shown that Foxo3a degradation is important for the survival of human memory CD4^+^ T cells [37], [38], but the effect of Foxo3a deficiency exclusively in antigen specific CD4^+^ T cells is largely unresolved and is complicated by the diverse biological pathways in which Foxo3a is an important master regulator.

Our observation that the role of Bim varied depending on the infection model indicates that a variety of factors may influence Bim activity in these settings. Our previous studies show that the survival of SMARTA following Lm-gp61 or LCMV infection is determined within the first five days of infection [14], suggesting that the role of Bim in promoting survival is not due to extended exposure to antigen or inflammation in the later stages of the response. Rather, we propose that qualitative differences in the nature of the activation signal in the early stages of the response are key. Because of the monoclonal nature of Bim-mediated elimination of SMARTA cells, our current hypothesis tests whether TCR signals play a key role in modulating Bim activity. However, this cannot fully explain the differences we see for the role of Bim between infections. One possibility is that differences in cytokines and/or activation environment may influence the impact of Bim during the CD4^+^ T cells response. The extent of inflammatory signaling is a key modulator of CD8^+^ memory T cells potential [39], and cytokines such as IL-2 or IL-21 may play a role in CD4^+^ T cell subset specification and subsequent memory development [40], [41]. A second intriguing possibility is that Bim differentially regulates the survival of different T helper subsets, such as Th1 (almost exclusively present during Lm-gp61 infection) and Tfh (the dominant effector subset following vaccinia virus infection). Regardless, it is clear that while Bim may respond to TCR signals, TCR-independent signals also likely influence its activity. Therefore, we find it unlikely that Bim functions solely to eliminate poorly functional responders. Instead, we propose that Bim plays a broad role in shaping the characteristics of emerging CD4^+^ memory T cells.
